# Euglycemic Diabetic Ketoacidosis Induced by Sodium-Glucose Cotransporter-2 Inhibitor Use and Coronary Angiography: A Case Report

**DOI:** 10.7759/cureus.52122

**Published:** 2024-01-11

**Authors:** Christopher D VonTungeln, Mohammad Al Bataineh

**Affiliations:** 1 Internal Medicine, University of Missouri Health Care, Columbia, USA; 2 Internal Medicine, University of Missouri Columbia, Columbia, USA

**Keywords:** stress-related cardiomyopathy, euglycemic diabetic ketoacidosis, endocrinology and diabetes, type 1 and type 2 diabetes mellitus, sodium-glucose cotransporter-2 (sglt-2) inhibitors

## Abstract

Euglycemic diabetic ketoacidosis (EDKA) is an uncommon subtype of diabetic ketoacidosis (DKA) which presents with similar laboratory findings to classic DKA with the exception of blood glucose levels being under 250 mg/dl. EDKA has several etiologies including pregnancy, starvation and the use of sodium-glucose cotransporter-2 inhibitors (SGLT-2). SGLT-2 inhibitors such as empagliflozin and dapagliflozin are increasing in popularity due to their positive benefits for patients with diabetes mellitus and cardiac disease. EDKA is underdiagnosed as it presents with blood sugar levels lower than expected in classic DKA. This case report describes a well-controlled type 2 diabetic patient prescribed an SGLT-2 inhibitor who developed EDKA after undergoing coronary angiography for acute heart failure.

## Introduction

Euglycemic diabetic ketoacidosis (EDKA) is a life-threatening complication of diabetes mellitus which is underdiagnosed [1]. EDKA is characterized by metabolic acidosis (arterial pH <7.3, serum bicarbonate <18mEq/L), ketosis (ketonemia and ketonuria), and blood glucose level <250 mg/dl. It may occur in type 1 or type 2 diabetes mellitus [2]. EDKA has several etiologies including decreased caloric intake, excessive alcohol consumption, pregnancy, and use of sodium-glucose cotransporter-2 inhibitors (SGLT-2) inhibitors [3]. 

SGLT-2 inhibitors, such as empagliflozin and dapagliflozin, are a relatively new type of medication that are FDA-approved for the treatment of type 2 diabetes mellitus and heart failure [4,5]. These medications act on the renal proximal convoluted tubules to reduce reabsorption of filtered glucose, decrease the renal threshold for glucose, and promote urinary glucose excretion [5]. EDKA has been rarely linked to SGLT-2 inhibitors and is more common in patients with type 1 diabetes or type 2 diabetes with insulin dependence [6]. EDKA accounts for 2.6% to 3.2% of diabetic ketoacidosis admissions and is increasing in incidence with the increase in use of SGLT-2 inhibitors [1]. 

We present a case of a well-controlled type II diabetic patient taking an SGLT-2 inhibitor who developed EDKA after coronary angiography was performed for acute heart failure. This case highlights the importance of clinical suspicion for EDKA in light of increasing rates of SGLT-2 inhibitor prescription rates.

## Case presentation

Our patient is a 54-year-old female with a past medical history of hypertension, fibromyalgia, chronic pain syndrome and type 2 diabetes mellitus. Her diabetes was well-controlled with a hemoglobin A1C level of 6.6% on the day of her hospital admission. She was taking metformin 1000 mg twice a day, pioglitazone 15 mg daily, and dapagliflozin 10 mg daily to manage her diabetes. She reported that she took insulin in the past but had not recently required insulin for hyperglycemic control. She had a spinal cord stimulator in place and had been getting frequent neural blockades for chronic pain management. Prior to admission she received a neural blockade from spinal level C7-T1 from an outpatient pain management clinic. Immediately after the procedure she developed shortness of breath, diffuse pain, and weakness of all four extremities. She was transported to the emergency room by ambulance. While in the ambulance her peripheral oxygen saturation (SpO2) was around 70% leading to initiation of bilevel positive airway pressure (BiPAP). 

On presentation to the emergency room the patient’s vitals were as follows: blood pressure of 127/96 millimeters of mercury (mmHg), pulse of 83 beats per minute, temperature of 36.0 degrees Celsius and SpO2 of 100% on BiPAP. A chest x-ray was performed which showed bilateral pulmonary opacities consistent with pulmonary edema. Initial complete blood count (CBC) and complete metabolic profile (CMP) were unremarkable. Electrocardiogram (EKG) showed left axis deviation and possible left atrial enlargement. A transthoracic echocardiogram was performed which showed an ejection fraction of 30% and findings consistent with stress-induced cardiomyopathy. She was admitted for further workup.

The following day she proceeded to undergo coronary angiography and was found to have no evidence of coronary artery disease. After coronary ischemia was ruled out the patient was scheduled for exchange of her spinal cord stimulator to a magnetic resonance imaging (MRI)-compatible device to obtain MRI of the thoracic spine. Routine labs (Table 1) and urinalysis (Table 2) were performed prior to the procedure. Basic metabolic panel (BMP) showed an anion gap of 29 mmol/L, CO2 of 17 mmol/L and glucose of 125 mg/dL. Urinalysis showed glucose >500 mg/dL and ketones of 80 mg/dl. Serum beta-hydroxybutyrate was then collected which was significantly elevated at >9.0mmol/L. Venous blood gas (VBG) was significant for pH of 7.157 with bicarbonate of 12.8 mmol/L. A diagnosis of EDKA was made. She was transferred to the intensive care unit for initiation of diabetic ketoacidosis (DKA) protocol with intravenous (IV) sodium chloride 0.9%, IV dextrose, and IV insulin. Her anion gap closed within 24 hours. She was then transitioned to subcutaneous insulin. She was transferred back to the floor for further management the following day.

**Table 1:** Significant hospital course serum laboratory results

	Day 1: Admission	Day 2: ICU transfer	Day 9: Discharge	Reference range
Sodium	142	140	138	136-145 mmol/L
Potassium	4.3	4.0	3.8	3.5-5.1 mmol/L
Chloride	106	94	100	98-107 mmol/L
CO2	27	17	27	22-29 mmol/L
Anion gap	9	29	11	0-20 mmol/L
Glucose (serum)	126	125	196	70-139 mg/dL
Beta-hydroxybutyrate	N/A	>9.0	N/A	0.10-0.27 mmol/L
pH (venous blood gas)	N/A	7.157	N/A	7.310-7.450
HCO3 (venous blood gas)	N/A	12.8	N/A	22.0-26.0 mmol/L
Hemoglobin A1c	6.6			4.0-5.6%

**Table 2:** Urinalysis results obtained prior to spinal cord stimulator exchange

	Day 2: ICU transfer	Reference range
Specific gravity	1.021	1.006-1.030
pH	5	4.5-8
Glucose	>500 glucose mg/DL	Negative
Ketones	80 mg/dL	Negative

After the patient received an MRI-compatible spinal cord stimulator, a thoracic spine MRI was obtained. She was diagnosed with an acute spinal cord injury as a result of the neural blockade she had received prior to admission. She was treated with an IV methylprednisolone taper. The etiology of the patient’s acute heart failure was attributed to stress-induced cardiomyopathy secondary to the spinal cord injury. Her symptoms continued to improve while inpatient and she was ultimately discharged to a post-acute care facility.

## Discussion

EDKA is a rare presentation of diabetic ketoacidosis with blood sugar levels under 250 mg/dL [2]. Carbohydrate deficiency has a significant role in the pathophysiology of EDKA compared to insulin deficiency or insulin resistance which is the driving force in classic DKA [1]. Counterregulatory hormone production is altered in EDKA leading to an increased glucagon/insulin ratio which triggers ketogenesis with minimal effect in hepatic glycogenolysis and peripheral glucose utilization explaining laboratory findings associated [1]. EDKA has been associated with fasting, pregnancy and the use of SGLT-2 inhibitors [6]. 

EDKA in the setting of SGLT-2 inhibitor use is much more common in patients with type 1 diabetes mellitus (9.4%) than type 2 diabetes mellitus (0.2%) [7]. A US FDA review of adverse events associated with SGLT-2 inhibitor use reported a fatality rate of 1.54% for EDKA compared to 0.4% for all DKA cases [7-9]. 

Patients with EDKA typically present with similar symptoms to classic DKA such as nausea, vomiting, malaise or fatigue [10]. Laboratory findings required to make the diagnosis of EDKA include metabolic acidosis with a pH less than 7.3, decreased serum bicarbonate less than 18 mEq/L, and increased serum and urine ketones in the setting of normoglycemia (capillary blood glucose less than 250 mg/dL) [2]. 

The patient’s SGLT-2 inhibitor should immediately be discontinued if EDKA is suspected [2]. Treatment is similar to classic DKA and consists of IV insulin, IV dextrose and IV fluid resuscitation until the anion gap and metabolic acidosis are corrected. IV fluid resuscitation is typically accomplished using normal saline or a balanced crystalloid solution such as Ringer’s lactate solution [11]. IV dextrose should be given the entire duration of the DKA protocol to avoid hypoglycemia due to near-normal blood glucose levels. Bicarbonate infusion is typically avoided, although may be considered if pH is less than 6.9 [2,12,13]. 

Criteria for resolution of ketoacidosis include blood glucose less than 200 mg/dl and two of the following criteria: a serum bicarbonate level greater than 15 mEq/L, a venous pH greater than 7.3, or a calculated anion gap equal or less than 12 mEq/L [12]. The patient should be transitioned to subcutaneous insulin after ketoacidosis has resolved with a one to two hour overlap between discontinuation of IV insulin and initiation of subcutaneous insulin to prevent recurrent hyperglycemia or ketoacidosis [13].

## Conclusions

EDKA is a rare and serious complication associated with the use of SGLT-2 inhibitors with findings suggestive of diabetic ketoacidosis in the setting of near-normal blood glucose levels. EDKA is underdiagnosed due to lower blood sugar levels at presentation when compared to classic DKA. Clinicians must be cognizant of the association of SGLT-2 inhibitors and EDKA as prescription rates continue to rise. Prompt diagnosis of EDKA will result in early treatment and improved outcomes in these patients.
